# Publisher Correction: Unveiling the Re effect in Ni-based single crystal superalloys

**DOI:** 10.1038/s41467-020-14820-0

**Published:** 2020-02-21

**Authors:** Xiaoxiang Wu, Surendra Kumar Makineni, Christian H. Liebscher, Gerhard Dehm, Jaber Rezaei Mianroodi, Pratheek Shanthraj, Bob Svendsen, David Bürger, Gunther Eggeler, Dierk Raabe, Baptiste Gault

**Affiliations:** 1 Max-Planck-Institut für Eisenforschung GmbH, 40237 Düsseldorf, Germany; 2 RWTH Aachen University, 52062 Aachen, Germany; 3 School of Materials, The University of Manchester, M13 9PL Manchester, UK; 4 Institut für Werkstoffe, Ruhr-Universität Bochum, Universitätsstrasse 150, D-44 780 Bochum, Germany; 5 Department of Materials, Royal School of Mine, Imperial College London, London, SW7 2AZ UK

**Keywords:** Mechanical properties, Metals and alloys

Correction to: *Nature Communications* 10.1038/s41467-019-14062-9, published online 20 January 2020.

The original version of this Article contained an error in Fig. 2h, in which the left-side label was given incorrectly as ‘(b3):SISF’, rather than the correct ‘(i):SISF’, and the right-side label was given incorrectly as ‘(c3):SESF’, rather than the correct ‘(j):SESF’.

The original version of this Article also contained an error in Fig. 8, in which all three *x*-axis tick marks were incorrectly labelled ‘200, 250, 300’. The correct *x*-axis tick marks read ’20, 25, 30’.

These errors have now been corrected in both the PDF and the HTML versions of the Article.

