# Supplementary material for: Unveiling the Re effect in Ni-based single crystal superalloys
Source: Nat Commun. 2020 Jan 20;11:389. doi: 10.1038/s41467-019-14062-9 (PMC6971021; doi:10.1038/s41467-019-14062-9)
Supplement: Supplementary file 1 — Supplementary Information [file 41467_2019_14062_MOESM1_ESM.pdf]

Supplementary information

Unveiling the *Re effect* in Ni-based single crystal superalloys

Wu et al.

Supplementary information 1
Supplementary Note 1 2
Supplementary Note 2 3
Supplementary Figures 5
Supplementary References 11

Supplementary Note 1

The average dislocation velocity $\langle v_d \rangle$ can be estimated using the Orowan's equation $\dot{\varepsilon} = b\rho_m \langle v_d \rangle$, where $\dot{\varepsilon}$ is the strain rate, b is Burger's vector magnitude, and ρ_m is the mobile dislocation density. In particular, at 1% creep strain, $\dot{\varepsilon} \approx 2.35 \times 10^{-6}\ s^{-1}$and $\rho_m = 0.9 \times 10^{13}\ m^{-2}$ (Ref. [1]) imply $\langle v_d \rangle \approx 1.78\ nm/s$. At 5% creep strain, the strain rate $\dot{\varepsilon} \approx 2.35 \times 10^{-8}\ s^{-1}$ is two orders of magnitude lower, resulting in a correspondingly lower average velocity estimate of $\langle v_d \rangle \approx 5.07 \times 10^{-3}\ nm/s$. A lower dislocation velocity increases the likelihood of more solute segregation, resulting in a reduction of the net dislocation glide force as indicated by the PF simulation results in Figure 8, and so a further velocity reduction.

Estimates in the literature (e.g., Ref. [2], Section 18-3) of the interaction force between a solute "cloud" or "Cottrell atmosphere" (i.e., concentration field) and a moving (edge) dislocation are based on many simplifying restrictions and assumptions not made in the phase field modelling discussed in the text. One of these is that only those solutes moving at the same velocity as the dislocation influence dislocation motion. In this context, the drag force (per unit length) of a Cottrell atmosphere moving together with a dislocation at velocity v_d is estimated as

$$f_d = v_d \frac{c_0 \beta^2}{DkT} \ln\left(\frac{R}{r_1}\right) \qquad (1)$$

((Ref. [2], Equation (18-60)). Here, $\beta = \frac{\mu b\ (1+v)\Delta v}{3\pi\ (1-v)}$ ((Ref. [2], Equation (14-47)), with μ the shear modulus, v Poisson's ratio, and Δv the difference in atomic volume (volume per atom) between the solute and solvent. In addition, R is the system size, $r_1 = \frac{\beta}{kT}$ ((Ref. [2], Equation (18-59)), c_0 the far-field (i.e., zero stress) solute number density (in atoms per unit volume), D the solute diffusivity, k Boltzmann's constant, and T the temperature. If $r_1$is smaller than the Volterra core cut-off radius r_0, it is replaced by the latter in (1). Under loading due to an external shear stress τ, combination of (1) with the overdamped velocity relation $v_d = m_d(\tau b - f_d)$ depending on the dislocation mobility m_d implies the result

$$v_d = \frac{m_d \tau b}{1 + m_d \frac{c_0 \beta^2}{DkT} ln\left(\frac{R}{r_1}\right)} \quad (2)$$

for the dislocation velocity. Note that (2) is insensitive to m_d when $m_d \gg \frac{DkT}{c_0 \beta^2 ln\left(\frac{R}{r_1}\right)}$ is tacitly assumed by Titus et al. (Ref. [3]) and others (Ref. [4,5]). To apply this to Re, assume for example $ln\left(\frac{R}{r_1}\right) = 2.1$ (Ref. [3,4,6]), $D = 1.67 \times 10^{-21} m^2/s$ (adapted from Ref.[7,8] for T=1023 K (750 °C)), 1.2 at.% Re implying $c_0 = 1.0 \times 10^{27}\ m^{-3}$, $\mu = 69\ GPa$, $v = 0.32$ (at 750 °C Ref.[9]), and $\Delta v = 1.306 \times 10^{30}\ m^3$. With τ of 377 MPa for the 5% creep strain samples, $v_d = 4.25 \times 10^{-2}\ nm/s$ is obtained from (2), assuming $m_d \gg \frac{DkT}{c_0 \beta^2 ln\left(\frac{R}{r_1}\right)}$. Similarly, a 14 at.% of Co and 9.5 at.% of Cr enriched at the dislocations yield dislocation velocities to be 53.86 nm/s and 76.35 nm/s, respectively. Re has a larger drag effect on dislocations than other solutes such as Cr, Co and W (Ref. [3,4,10]). Thus, under the same external load, dislocations decorated with Re will move slower, therefore could represent the rate-limiting factor on creep. Note that the concentration used in the equation (2) is the amount of enrichment in the partial dislocations in the γ' phase obtained by APT. The far-field concentration is also utilized for estimation, which shows the same trend, i.e., Re is the slowest moving element compared to Cr, Co and W.

Supplementary Note 2

Estimation of Re atoms transported:

Parameters from previous studies [1]

γ' volume fraction	73%
Typical γ' cube edge	442 nm
Typical γ channel length	65 nm
Dislocation density in γ	25×10^{13} m^{-2}
Dislocation density in γ' (5% strain)	3×10^{13} m^{-2}
Overall Re concentration	0.9 at.%
Re concentration in γ	3.3 at.% (in equilibrium at 750°C, from ThermoCalc)

Re concentration in γ'	0.07 at.% (in equilibrium at 750°C, from ThermoCalc)
Typical Re concentration in PD	1.2 at.%
Lattice constant *a*	0.365 nm

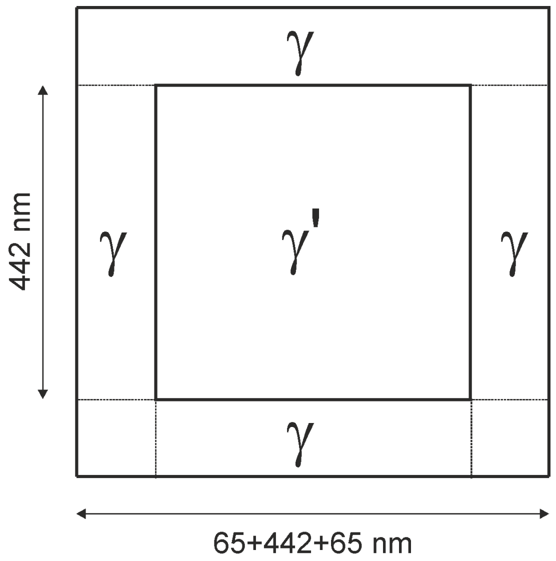

The area fraction of γ

$A_{\gamma} = 1.32\times10^{-13}\,m^2$

Overall area fraction of γ: $A_{\gamma\_overall} = 0.36\times10^{-13} m^2$

<u>**Re atoms in γ**</u>: $\frac{3.3\,at.\%}{0.36\times10^{-13}m^2} = 9.17\times10^{13}\frac{at.\%}{m^2}$

The area fraction of γ'

$A_{\gamma'} = 1.95\times10^{-13} m^2$

Overall area fraction of γ: $A_{\gamma'\_overall} = 1.42\times10^{-13}\,m^2$

<u>**Re atoms in γ':**</u> $\frac{0.07\,at.\%}{1.42\times10^{-13}m^2} = 0.049\times10^{13}\frac{at.\%}{m^2}$

<u>**Re atoms transported to γ' by partial dislocation at 5% strain**</u>, under the assumption that all partial dislocation inside of γ' carry the same maximum amount of Re:

1.2 at. % × $3\times10^{13}\,m^{-2} = 3.6\times10^{13}\frac{at.\%}{m^2}$

Under this extreme assumption, approximate 1/3 of the Re atoms were transported from the γ channel to the γ' phases.

Supplementary Figures

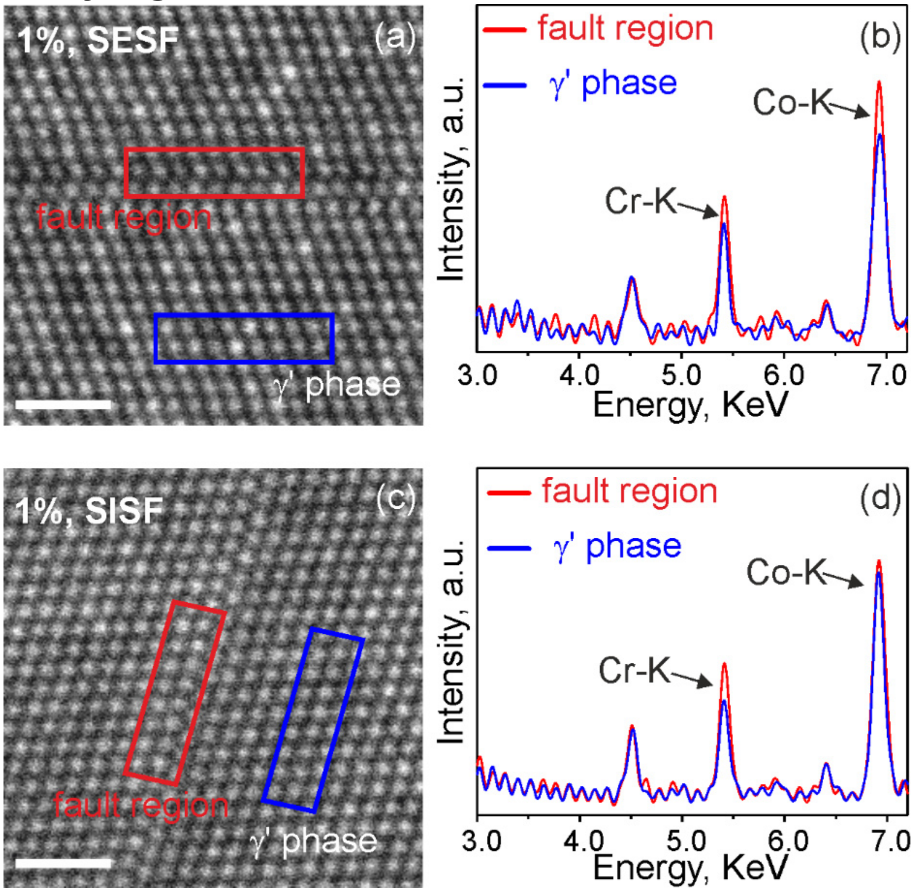

Supplementary Figure 1: Comparison of spectrum between fault region and the precipitate region from STEM EDS mapping for SISF and SESF in the specimen crept at 1%. The spectrum is normalised with Ni kα peak intensity. (a) HAADF for SESF of 1% crept specimen. (b) Comparison of spectrum for SESF in (a) showing higher intensity of Cr and Co in the fault region. (c) HAADF for SISF of 1% crept specimen. (d) Comparison of spectrum for SISF in (a) showing higher intensity of Cr and Co in the fault region. Scale bar: 1 nm.

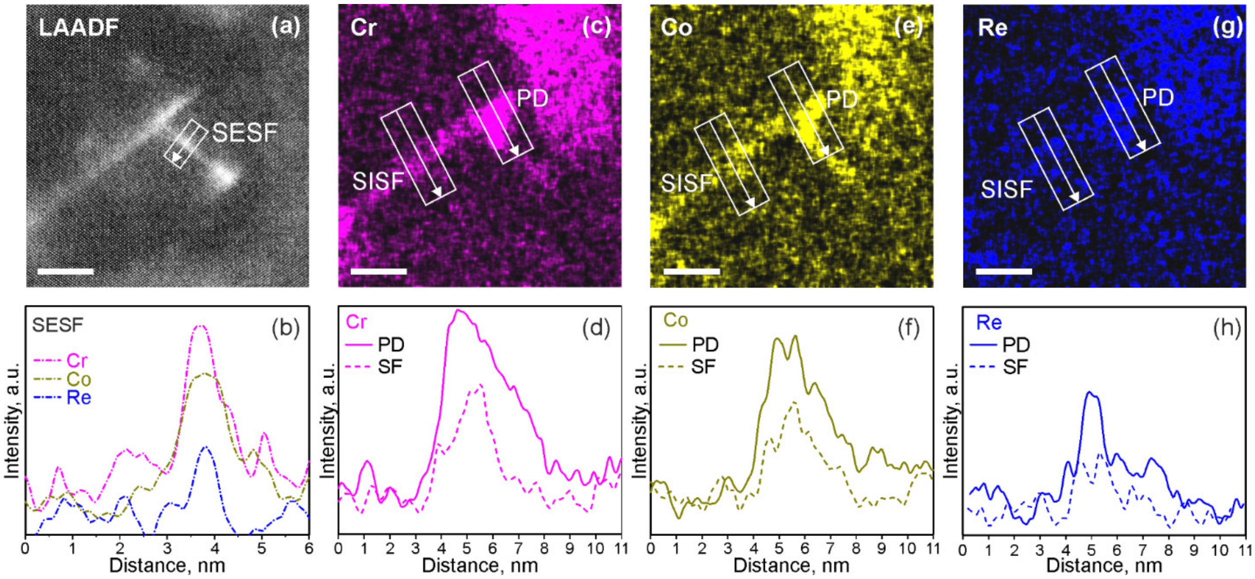

Supplementary Figure 2: (a) LAADF for a PD, a SISF, and a SESF of 5% crept specimen. The white rectangle indicates the region of line intensity integration area, following the direction of the arrow. (b) Comparison of elemental intensity for the SESF in the area from (a), showing a higher intensity of Cr, Co and Re in the fault region. (c) EDS mapping for Cr, two rectangles showing regions for line intensity integration for PD and SISF. (d) Comparison of integrated intensity of Cr for PD and SISF regions, indicating higher concentration of Cr in the PD region compared to the SISF. (e) EDS mapping for Co, two rectangles showing regions for line intensity integration for PD and SISF. (f) Comparison of integrated intensity of Co for PD and SISF regions, indicating higher concentration of Co in the PD region compared to the SISF. (g) EDS mapping for Re, two rectangles showing regions for line intensity integration for PD and SISF. (h) Comparison of integrated intensity of Re for PD and SISF regions, indicating higher concentration of Re in the PD region compared to the SISF. Scale bar: 5 nm.

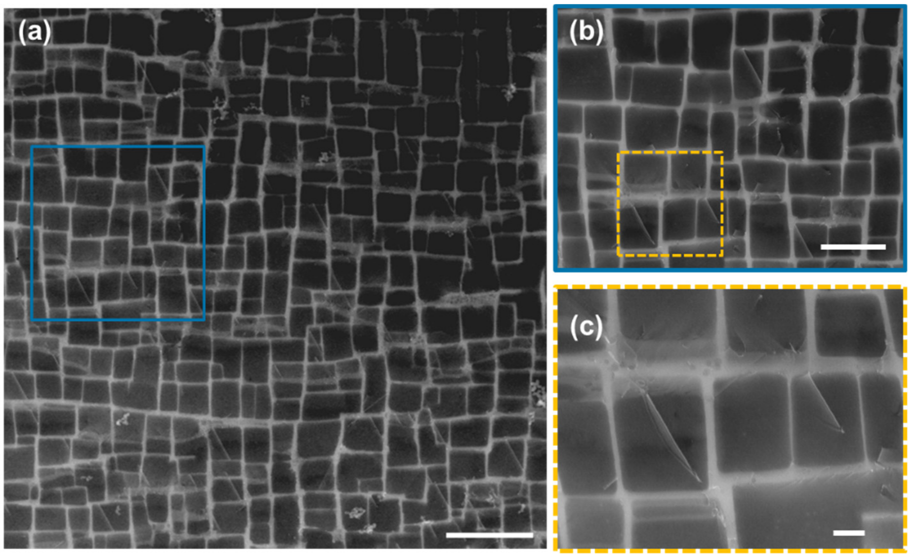

Supplementary Figure 3: ECC images showing SF in 1% creep sample for correlative TEM/APT sample preparation. Scale bar: (a) 2 µm, (b) and (c): 500 nm.

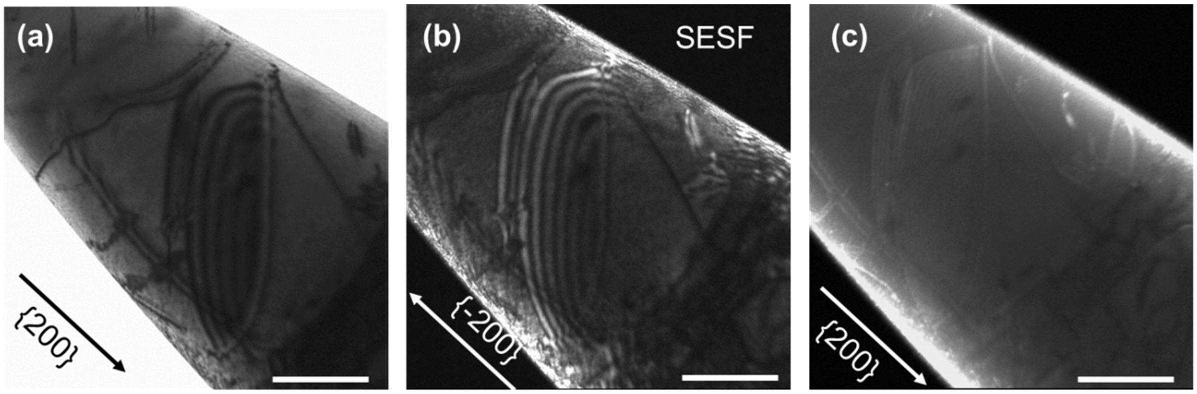

Supplementary Figure 4: TEM images of a correlative APT/TEM tip for 1% creep specimen containing dislocations and SFs. (a) BF image. (b) CDF (centered dark field) image. (c) WBDF (weak beam dark filed) image. All scale bars: 200 nm.

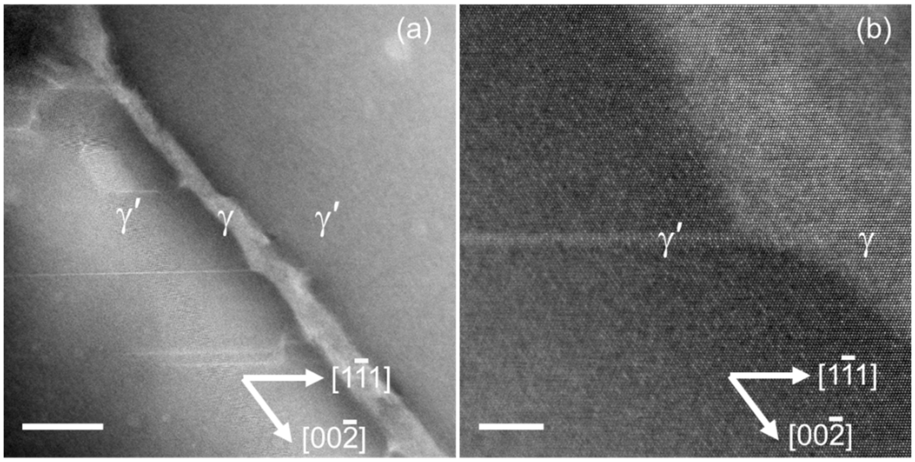

Supplementary Figure 5：STEM HAADF images for 5% crept specimen showing serrated g/g' interface. (a) Low magnification showing several serrated positions at the g/g' interface with SFs. (b) enlarged view showing one example of the SSF connecting with the serrated position. Scale bar in (a): 50 nm; (b) 5 nm.

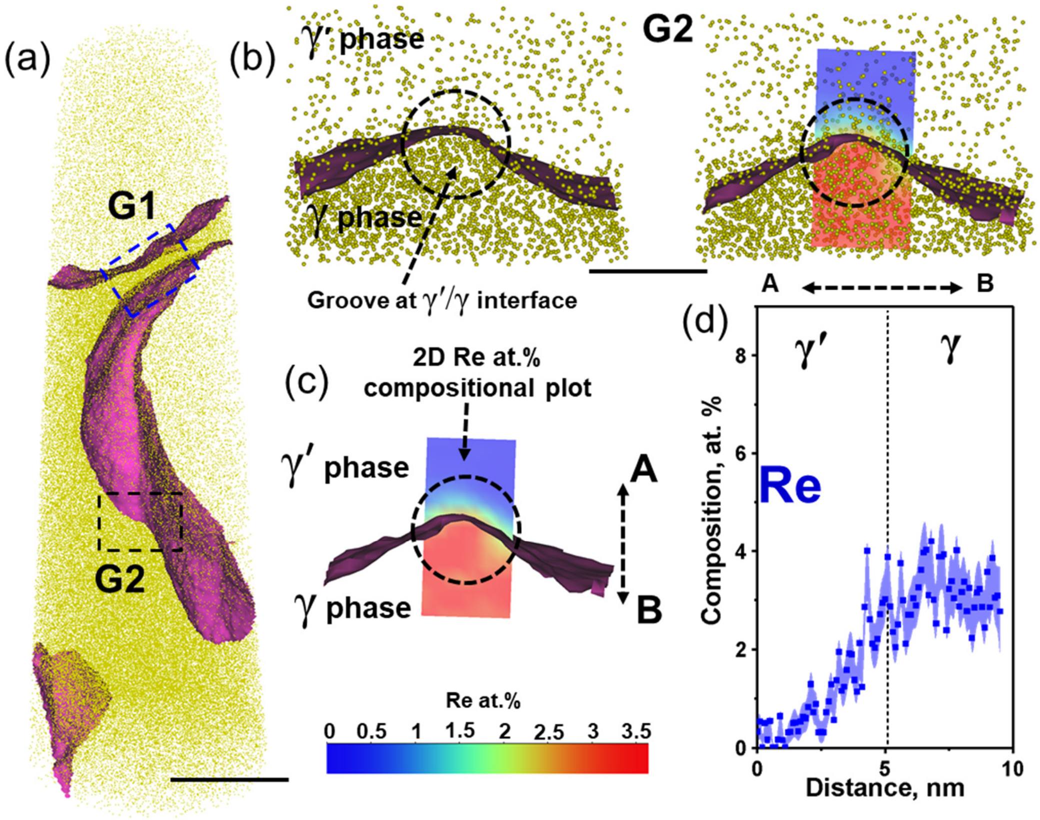

Supplementary Figure 6 : APT analysis of grooves at the interface for 5% crept sample. (a) APT reconstruction showing γ/γ' interfaces and two groove regions G1 and G2. The detailed analysis of groove G1 is shown in Figure 5. (b) Enlarged view of groove region G2 showing no enrichment of Re at the interface groove without dislocation. (c) Clean view showing the γ/γ' interface confirming no enrichment at the groove, AB shows the direction for 1D compositional analysis in (d). (d) 1D compositional profile of Re through the interface dislocation. Scale bar: (a) 50 nm. (b) 20 nm. The error bars in Supplementary Figure 6(d) are estimated using the equation in Methods section

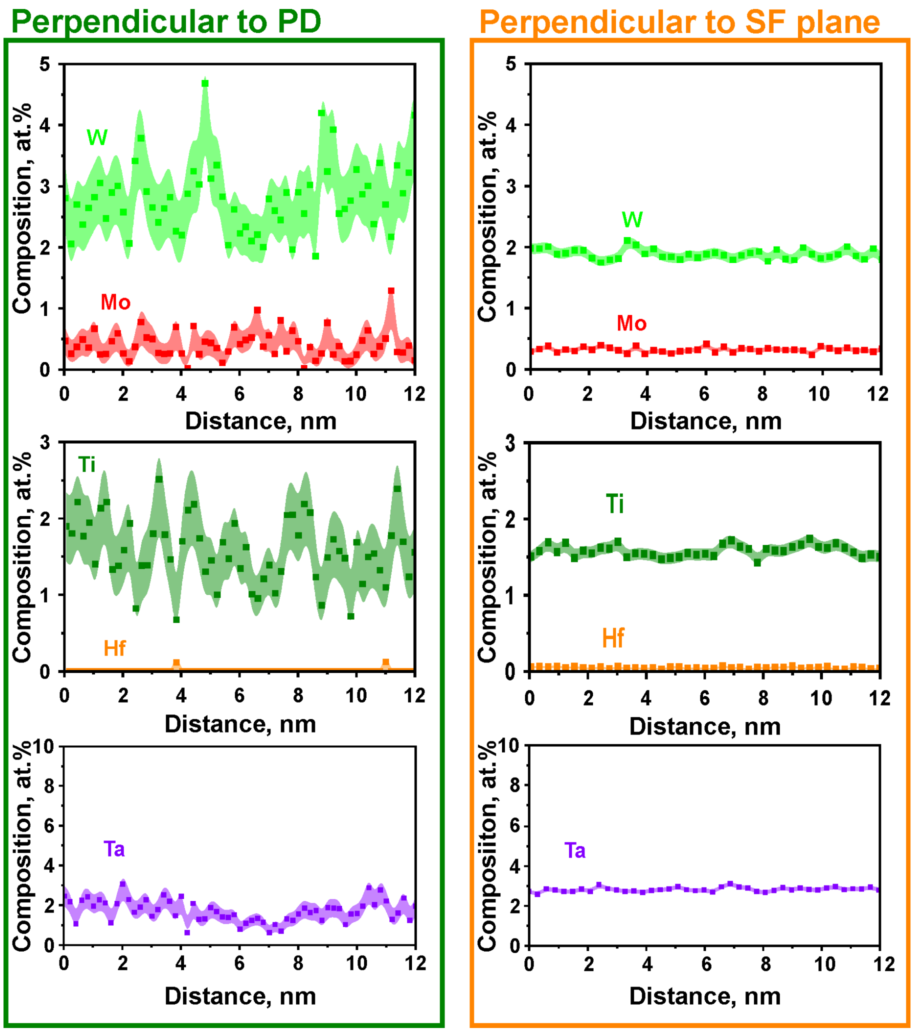

Supplementary Figure 7: APT analysis for 5% creep specimen, 1D compositional profile showing the distribution of the elements (W, Mo, Ti, Hf, and Ta) in the direction: left panel - perpendicular to PD; right panel- perpendicular to SF plane. The error bars in Figure 7 are estimated using the equation in Methods section

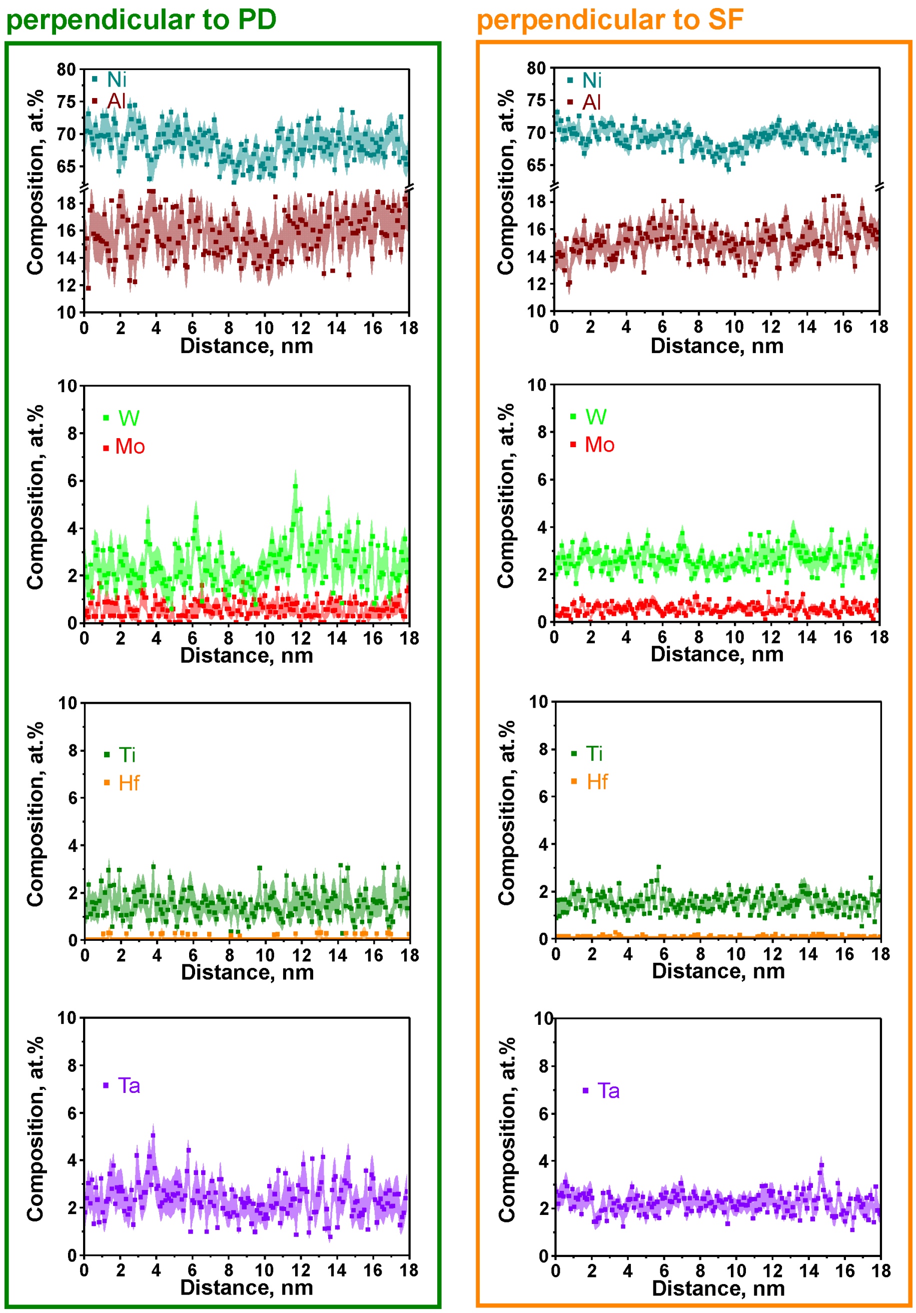

Supplementary Figure 8: APT analysis for the ruptured specimen, 1D compositional profile showing the distribution of the elements (Ni, Al, W, Mo, Ti, Hf and Ta) in the direction: left panel - perpendicular to PD; right panel- perpendicular to SF plane.

Supplementary References

1. Wu, X. *et al.* Double minimum creep of single crystal Ni-base superalloys. *Acta Mater.* **112**, 242–260 (2016).

2. Hirth, J. P. & Lothe, J. Theory of dislocations. (1982).

3. Titus, M. S. *et al.* High resolution energy dispersive spectroscopy mapping of planar defects in L12-containing Co-base superalloys. *Acta Mater.* **89**, 423–437 (2015).

4. Makineni, S. K. *et al.* On the diffusive phase transformation mechanism assisted by extended dislocations during creep of a single crystal CoNi-based superalloy. *Acta Mater.* **155**, 362–371 (2018).

5. Smith, T. M. *et al.* Effect of stacking fault segregation and local phase transformations on creep strength in Ni-base superalloys. *Acta Mater.* **172**, 55–65 (2019).

6. Smith, T. M. *et al.* Segregation and η phase formation along stacking faults during creep at intermediate temperatures in a Ni-based superalloy. *Acta Mater.* **100**, 19–31 (2015).

7. Hargather, C. Z., Shang, S.-L. & Liu, Z.-K. A comprehensive first-principles study of solute elements in dilute Ni alloys: Diffusion coefficients and their implications to tailor creep rate. *Acta Mater.* **157**, 126–141 (2018).

8. Hargather, C. Z., Shang, S. & Liu, Z. K. Data set for diffusion coefficients and relative creep rate ratios of 26 dilute Ni-X alloy systems from first-principles calculations. *Data Br.* **20**, 1537–1551 (2018).

9. W. Hermann; H.G. Sockel; J. Han; A. Bertram. Elastic properties and determination of elastic constants of Nickel-base superalloys by a free-free beam technique. in *Superalloys 1996* 229–238 (1996).

10. Smith, T. M., Rao, Y., Wang, Y., Ghazisaeidi, M. & Mills, M. J. Diffusion processes during creep at intermediate temperatures in a Ni-based superalloy. *Acta Mater.* **141**, 261–272 (2017).
